# Salvage treatment for lymph node recurrence after radical resection of esophageal squamous cell carcinoma

**DOI:** 10.1186/s13014-019-1377-y

**Published:** 2019-09-18

**Authors:** Jie Chen, Wenming Yin, Hui Yao, Wendong Gu

**Affiliations:** Department of Radiation Oncology, The Third Affiliated Hospital of Soochow University, No. 185 Juqian Street, Changzhou, 213003 China

**Keywords:** Esophageal squamous cell carcinoma, Radical surgery, Lymph node recurrence, Radiotherapy, Chemoradiotherapy

## Abstract

**Background:**

Patients with regional lymph node recurrence after radical resection of esophageal cancer have poor therapeutic outcomes. Currently, there is no standard treatment for regional lymph node recurrence, and its prognostic risk factors are not well-understood. This study retrospectively analyzed 83 patients with postoperative regional lymph node recurrence after radical resection of esophageal squamous cell carcinoma. The aim was to evaluate the clinical efficacy and prognostic factors of salvage radiotherapy with or without chemotherapy in these patients.

**Methods:**

The survival and prognostic factors of 83 patients with esophageal squamous cell carcinoma with regional lymph node recurrence after radical surgery were retrospectively analyzed. All patients underwent radiotherapy, of which 74 patients received volumetric modulated arc therapy (VMAT), 9 patients received three-dimensional conformal radiation therapy (3DCRT), administered using a conventional segmentation protocol with a dose distribution range of 50.4–66.2Gy (median dose of 60Gy). In total, 41 patients received radiotherapy alone, 42 received radiotherapy combined with chemotherapy, and the concurrent chemotherapy regimen was mainly composed of either platinum or fluorouracil monotherapy, except for 4 patients who were given 5-fluorouracil plus platinum (FP) or paclitaxel plus platinum (TP).

**Results:**

The median follow-up time was 24 (range, 9–75) months. The overall survival (OS) rates at 1 year, 2 years, 3 years, and 5 years were 83.0, 57.1, 40.1, and 35.1%, respectively. The median overall survival (OS) time was 18 (range, 5–75) months. The 3-year survival rate was 47.5% in patients with radiation alone and 41.9% in patients receiving concurrent chemoradiotherapy(*p* = 0.570), while the response rate (CR + PR) in those two groups was 73.2 and 91.4%, respectively. By multivariate analysis of OS, age (worse in younger patients, *p* = 0.034) was found to be significantly associated with disease prognosis. The commonly toxicities were esophagitis, neutropenia and anemia. 18% patients experienced grade 3 toxicity and no treatment-related death occurred.

**Conclusions:**

These results of this retrospective analysis suggest that radiotherapy with or without chemotherapy is an effective and feasible salvage treatment for lymph node recurrence after radical resection of esophageal squamous cell carcinoma.

## Introduction

In China, surgical resection is the preferred treatment for resectable esophageal cancer. However, the 5-year overall survival rates of these patients are low, ranging from 31 to 55% [1, 2]. In addition, 43%~ 53% of patients who undergo surgery experience local recurrence and/or distant metastasis, and the median time to recurrence ranges from 10 to 12 months. The most common local recurrence sites are anastomotic, supraclavicular lymph nodes and mediastinal lymph nodes [1, 3, 4]. Treatments for local recurrence include chemoradiotherapy, radiotherapy, surgery, chemotherapy and stringent supportive care. A retrospective study by Kosuga et al. [5]indicated that lymphadenectomy, radiotherapy, and chemoradiotherapy were effective salvage treatments. Lymphadenectomy has been reported to be feasible for postoperative cervical lymph node recurrence [6–8]. Radiotherapy administered locally was also found to be a promising option to control lymph node recurrence after curative resection [9–11]. Given that chemoradiotherapy is effective in mounting local control and reducing distant recurrence by promoting radiosensitivity, while eliminating micrometastases, several studies have verified the effectiveness of concurrent chemoradiotherapy [12–14]. However, the prevailing data on this treatment is not conclusive and many therapeutic strategies for postoperative lymph node recurrence after esophageal cancer are controversial. Herein, we determined the prognostic factors of patients with esophageal squamous cell carcinoma with regional lymph node recurrence after radical surgery and the efficacy of radiotherapy with or without chemotherapy for lymph node recurrence in such patients.

## Materials and methods

### Study population

We retrospectively analyzed the survival and prognosis factors of patients who underwent radiation therapy for postoperative regional lymph node recurrence after resection of esophageal cancer in the Third Affiliated Hospital of Suzhou University from January 2012 to January 2018. Data was obtained from medical records, radiotherapy plans and video material. A total of 83 patients with lymph node recurrence following curative resection of esophageal squamous cell carcinoma were enrolled in the study, and all patients provided informed consent. The inclusion criteria included: (1) pathology confirmed as esophageal squamous cell carcinoma; (2) initial treatment was radical surgery without neoadjuvant therapy or postoperative prophylactic radiotherapy; (3) lymph node recurrence was diagnosed by 2 senior physicians experienced in imaging or pathological data evaluation, including the supraclavicular, mediastinal and left gastric lymphatic drainage area; (4) Kanofsky Performance Status (KPS) ≥ 70; (5) neither anastomotic recurrence nor distant metastasis was present after CT examination at this hospital before radiotherapy; (6) diagnosis criteria of lymph node recurrence: 1) CT showed the shortest diameter of the suspicious lymph node ≥1 cm, the longest diameter of the lymph nodes beside the esophagus, at the corner between the esophagus and trachea, or at the cardio-diaphragmatic angle > 5 mm, and the longest diameter of abdominal lymph node ≥5 mm [15–17]; or 2) CT showed more than 3 lymph node clustered or lymph nodes fused to each other; or 3) CT showed lymph node center necrosis; or 4) Positron Emission Tomography–Computed Tomography (PET/CT) showed high FDG uptake lesions in the esophageal lymph node drainage area (SUV cut-off value > 2.5 [18–20]); or 5) puncture pathology confirmed the existence of lymph node metastasis.

### Treatment

All patients received intensity-modulated radiation therapy (VMAT and 3DCRT radiotherapy). Patients were placed in supine position, a cradle for immobilization was made with vacuum (Gray) followed by body membrane fixation (Klarity) and spiral CT positioning (Siemens Somatom Sensation Open). Each patient was scanned from the axis (C2) to the second lumbar vertebrae (L2) level to cover the entire neck, lung, esophagus and abdominal lymph node regions. Images were reconstructed to 5 mm thick after CT enhancement scanning and transferred to the Treatment Planning System (TPS) to determine the target area and radiation therapy plan, and to optimize the measurement. Target area delineation: The gross tumor volume (GTV) was defined as recurrent lymph nodes identified by CT scans or PET/CT. The clinical target volume (CTV) was defined as GTV plus a margin of 10 mm around GTV and manually modified to meet the following criteria: 1) include the nearest adjacent lymphatic drainage regions of the metastasis lymph node; 2) avoid normal organs such as the pleura, vertebral bodies, and blood vessels. The planning tumor volume (PTV) was defined as the CTV plus the placement of 5 mm around the CTV. Both the 3D-CRT and VMAT treatment plans were designed by the Monaco Radiotherapy Treatment Planning System (Elekta). Radiation therapy (RT) was administered with a linear 6-MV photon accelerator (Elekta Axesse). The daily fractional dose of RT was 1.8–2.0Gy, administered 5 days per week, and the total dose was 50.4–66.2Gy. The dose of OAR was administered as follows: maximum dose of spinal cord<45Gy, mean heart dose <35Gy, lung doseV20 < 30%. Response evaluation was assessed 1 month after radiation treatment. Patients were followed up at intervals of 3 to 6 months after treatment. The response assessment included medical history, ultrasonography, chest and abdomen CT and PET/CT. Response Evaluation Criteria in Solid Tumors (RECIST 1.1) were used to determine tumor response, and infield disease progression was defined as treatment failure.

### Statistical analyses

Overall survival (OS) time was calculated as the time interval from the initiation of radiotherapy to death or termination of follow-up after relapse. The OS rate was estimated by Kaplan-Meier method and the differences in survival rates after univariate analysis were assessed with the log-rank test. Factors with p<0.2 were included in multivariate analysis. Cox’s proportional hazards regression model was used for multivariate survival analysis. All statistical analyses were performed using SPSS 21.0 software. *P* values < 0.05 were regarded as statistically significant. Short-term efficacy was assessed based on the solid tumor efficacy evaluation criteria (RECIST 1.1). The tumor response was defined as a complete response (CR) and a partial response (PR). Toxicity was assessed and documented according to National Cancer Institute Common Terminology Criteria for Adverse Events (CTCAE 4.1).

## Results

### Patients and tumor characteristics

A total of 83 patients were eligible for this study, and their clinical characteristics are listed in Table 1. All patients completed radiotherapy, 41 patients received radiotherapy alone, 42 patients received radiotherapy combined with chemotherapy and 35 of the 42 patients received concurrent chemoradiotherapy. The median radiation dose was 60Gy (range, 50.4 to 66.2Gy). A total of 46 patients received radiation above the 60Gy dose and 37 received radiation of less than 60Gy. Seventy-four patients used VMAT technology and nine patients used 3D-CRT technology. All patients received regular follow-up after treatment. The median follow-up time was 24 months (range, 9–75 months). The median time from surgery to diagnosis of regional lymph node recurrence was 5 months (range, 1–59 months). The recurrent lymph nodes were mainly distributed in the mediastinum and supraclavicular regions. Six cases had supraclavicular lymph node recurrence, 50 cases had mediastinal lymph node recurrence, 2 cases had abdominal lymph node recurrence and the remaining 25 cases had multi-regional lymph node metastasis of which 23 cases experienced supraclavicular and mediastinal lymph node recurrence. The median volume of the recurrent tumor was 5.5cm^3^ (range, 1.22–123.21cm^3^). According to previous works investigating the prognostic factors, age, postoperative stage of esophageal cancer, interval to recurrence, lymph node recurrence sites, number of recurrent nodes, recurrent lymph node size, with or without concurrent chemotherapy, radiation dose, chemotherapy regimen and tumor response rate were included in the univariate analysis to evaluate their impact on prognosis. The cutoff values of the factors including age, recurrent lymph node size and radiation dose were decided according to the median.

**Table 1:** Clinical characteristics of patients (*n* = 83)

Characteristic	Group	No.	(%)
Age (year)	Median (range)	63 (45–76)	
	≥63	43	(52)
	<63	40	(48)
Gender	Male	67	(81)
	Female	16	(19)
Performance Status (KPS)	≥80	78	(94)
	70–80	5	(6)
Primary tumor location	Cervix+Upper thorax	6	(7)
	Middle thorax	36	(43)
	Lower thorax	41	(49)
Radical surgery	Ivor-Lewis	48	(58)
	Sweet	26	(31)
Stage of primary tumor	I + II	49	(59)
(AJCC 7th edition)	III	34	(41)
Interval to recurrence (month)	Median (range)	5 (1–59)	
	>6	20	(24)
	≤6	63	(76)
Lymph node recurrence site	Supraclavicular region	6	(7)
	Mediastinal	50	(60)
	Abdominal	2	(2)
	Multiple	25	(30)
Recurrent lymph node size (cm^3^)	Median (range)	5.5 (1.22–123.21)	
	>5.5	41	(49)
	≤5.5	42	(51)
Number of lymph node recurrence (no.)	Median (range)	2 (1–6)	
	Multiple	64	(77)
	Single	19	(23)
Radiation dose	Median (range)	60 (50.4–66.2)	
	≥60Gy	46	(55)
	<60Gy	37	(45)
Treatments after recurrence	RT^a^	41	(49)
	RT + CT^b^	42	(51)

^a^radiotherapy alone

^b^radiotherapy combined with chemotherapy

### Treatment outcome

**Fig. 1:**
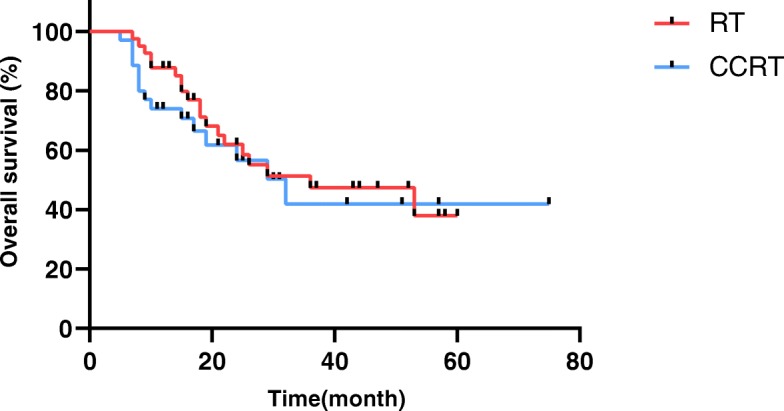
Overall survival of patients treated with radiotherapy alone and concurrent chemoradiotherapy. The median OS and the 1 years,3 years OS rates were 22 months, 79.8, and 47.5% in the group of patients treated with radiotherapy alone (RT), while that of 16 months, 74.1 and 41.9% in cases with concurrent chemoradiotherapy (CCRT). In the univariate analysis of OS assessed with the log-rank test, there was no significant differences between the two group (*p* = 0.570)

The overall tumor response rate was 81.9%, with 10 patients showing complete response (CR) and 58 patients showing partial response (PR) (Table 2). The 1-year, 2-year, and 3-year, 5-year OS rates were 83.0, 57.1, 40.1, and 35.1%, respectively. The median OS was 18 months (range, 5–75 months). In the radiotherapy group alone, the 3-year survival rate, the response rate and the median OS were 47.5, 73.2% and 22 months, respectively, while the 3-year survival rate, the response rate and the median OS were 41.9, 91.4% and 16 months, respectively, in patients who received concurrent chemoradiotherapy (Fig. 1).

**Table 2:** Tumor response rate

Tumor response	Total (*n* = 83) No. (%)		RT (*n* = 41) No. (%)		CCRT (*n* = 35) No. (%)	
CR^a^	10	(12.0)	5	(12.2)	5	(14.3)
PR^b^	58	(69.9)	25	(61.0)	27	(77.1)
SD^c^	9	(10.9)	7	(17.1)	2	(5.7)
PD^d^	6	(7.2)	4	(9.7)	1	(2.9)

^a^complete remission

^b^partial remission

^c^stable disease

^d^progressive disease

### Toxicity

The main toxicities were esophagitis, neutropenia and anemia, and most of the toxicity were of grade 1 or 2. Grade 3 toxicities were observed in 15 patients, including: skin damage (1.2%), nausea and vomiting (1.2%), bronchitis (1.2%), neutropenia (4.8%), radiation pneumonitis (9.6%). Patients with high dose (≥60Gy) or radiotherapy combined with chemotherapy were more likely to have grade 3 toxicity. No grade 4 or 5 toxicity was observed in any patients, and no treatment-related death occurred.

### Statistical analysis

In the univariate analysis of OS, no statistically significant factors were found to be associated with disease prognosis. In the multivariate analysis of OS, age (worse in younger patients, *P* = 0.034) was characterized as a significant independent prognostic factor. (Table 3).

**Table 3:** Univariate and multivariate analysis of the overall survival prognostic factors (*n* = 83)

Variable	Univariate analysis			multivariate analysis	
	No.	χ^2^	*P*	*HR((95%CI)*	*P*
Gender					
Male	67	1.689	.194		
Female	16			.584(.170–2.008)	.394
Age					
≥ 63 years	43	1.782	.182		
<63 year	40			2.345 (1.068–5.147)	.034
Primary tumor location					
Cervix+Upper thorax	6	2.061	.357		
Middle thorax	36				
Lower thorax	41				
Radical surgery					
Ivor-Lewis surgery	48	.385	.535		
Sweet surgery	26				
pT					
pT1 + 2	35	.036	.850		
pT3 + 4	48				
pN					
pN0	40	1.769	.184		
pN+	43			1.339(.622–2.879)	.456
pStage^a^(AJCC 7th edition)					
I + II	49	1.456	.228		
III	34				
Interval to recurrence					
>6 months	20	.001	.980		
≤ 6 months	63				
Recurrent lymph node size					
>5.5 cm^3^	41	1.146	.284		
≤ 5.5 cm^3^	42				
Number of recurrent nodes					
Multiple	64	.607	.436		
Single	19				
Region of recurrent nodes					
Multiple	25	1.104	.293		
Single	58				
Lymph node recurrence site					
Supraclavicular	6	4.586	.101		
Mediastinal	50			1.839(.430–7.856)	.411
Abdominal	2			3.123 (.262–37.160)	.367
Salvage Treatments					
RT	41	.322	.570		
CCRT^b^	35				
Radiation technique					
VAMT^c^	74	.248	.618		
3DCRT^d^	9				
Concurrent chemotherapy regimen					
platinum	18	.801	.371		
fluorouracil	15				
Radiation dose					
≥ 60Gy	46	.020	.888		
<60Gy	37				
Tumor Response					
CR + PR	68	.331	.565		
SD + PD	15				

^a^postoperative stage of esophageal cancer

^b^concurrent chemoradiotherapy

^c^volumetric modulated arc therapy

^d^three-dimensional conformal radiation therapy

## Discussion

Our results showed that radiotherapy, with or without chemotherapy, is an effective and feasible salvage treatment for lymph node recurrence after radical resection of ESCC. The 1-year, 3-year, and 5-year OS rates were 83.0, 40.1, and 35.1%, with the median OS of 18 months. In the radiotherapy group, the 3-year survival rate was 47.5%, the response rate was 73.2% and the median OS was 22 months. In patients who received concurrent chemoradiotherapy, the 3-year survival rate, the response rate and the median OS were 41.9, 91.4% and 16 months, respectively. Grade 3 toxicity was low (18%) and there were no treatment-related deaths that occurred. In the multivariate analysis of OS, age was characterized as a significant independent prognostic factor(*P* = 0.034). Similar to previous studies, our results are robust. In the study of Ma et al. [10], 98 patients were randomly enrolled to undergo either three-dimensional conformal radiotherapy alone (group A) or concurrent chemoradiotherapy (group B). All patients received a radiation dose of 62-70Gy and the patients in group B received a weekly low dose of cisplatin (30 mg/m^2^). The median OS and 3-year survival rates of group B (35 months and 46.9%, respectively) were greater than those of group A (19 months and 28.6%, respectively). Our overall 3-year OS rates (47.5%) were greater than those (28.6%) reported by Ma et al. [10] in the RT group, while they were similar in the CCRT group. In addition, researchers found that neither of the treatment modalities brought about any improvements to the 5-year survival rates. VMAT was commonly used in our study. Clinical research and methodological studies relating to the treatment of esophageal cancer have shown that IMRT or VMAT are better than 3DCRT with respect to improved target coverage and conformality, in addition to reduced radiation exposure to adjacent organs [25, 26].This may be the reason why our results were favorable. Previous studies suggested that synergistic effects of concurrent chemoradiotherapy can improve survival. Jingu et al. [24] reported the long-term results of CCRT for postoperative lymph node recurrence in their prospective phase II study. A total of 30 patients were treated for post-operative LR with RT (60Gy) combined with concurrent chemotherapy consisting of two cycles of nedaplatin and 5-fluorouracil. The 3-year OS was 38.4%, with an MST of 21.0 months. Yamashita et al. [22] also reported similar findings in a study that involved 237 patients who received RT or CCRT. The 3-year OS was 39.7% with CCRT and 20.8% with RT alone (p<0.05). In her study, 83% of patients received CCRT, among which, 5-fluorouracil and cisplatin/ nedaplatin were used in 167patients. In the present study, Platinum or fluorouracil monotherapy were predominantly used; it may reduce the toxicity compared to reported studies. Despite the controversy in concurrent chemotherapy regimens that are suitable for postoperative lymph node recurrence, the FP regimen produced excellent results for recurrent lymph nodes reported by Zhang et al. [13] Studies on CCRT for patients with locoregional recurrent esophageal carcinomas are limited in countries with the exception of Asia. Baxi el at [27]. treated 14 patients with both adenocarcinoma and SCC. All patients received 58–60Gy, with either cisplatin, fluorouracil, or both. Similar to our results, the median OS was 16 months and the 2-year OS was 21%. Jeene el at [28]. investigated salvage treatment for an isolated lymph node recurrence after curative resection in 22 patients. Treatment consisted of 50.4Gy combined with weekly concurrent paclitaxel and carboplatin therapy. The study reported a median OS was 33 months, which, is better than the findings from the present study. In western countries, it is more common to use neoadjuvant chemoradiotherapy followed by surgery as a standard for the radical treatment of esophageal cancer. However, the dose or the area of re-irradiation may be limited, especially on those infield recurrences. In those studies, adenocarcinomas were mostly the cancers effecting the participants. Although, there was no agreement on prognosis between adenocarcinomas and SCC. In addition, the recurrence at the site of the anastomosis has been reported prognostically unfavorable in those two studies. In this study, the differences in recurrent lymph node size might have been one reason for the difference in the survival periods. The median size of 8.14cm^3^ in the CCRT group was significantly greater than that of 4.15 cm^3^ in the radiotherapy group. Previous studies stated that small recurrent lymph nodes have a better prognosis [10]. There was no other difference between the two groups, including radiation dose (median 60Gy in both groups), interval to recurrence, region of lymph node recurrence, and postoperative staging. Controversies remain regarding the treatment strategy for lymph node recurrence after radical resection in esophageal cancer. Radiotherapy or chemoradiotherapy may be selected as salvage treatment considering the good survival results and low toxicity reported in this study. However, lymph node recurrence, anastomotic recurrence, and distant metastasis occurred even after salvage treatment. Given the presence of micrometastasis, further prospective studies should be carried out to determine populations that have high risk of metastasis so that they may receive intensive therapy.

As for prognostic factors, age was characterized as a significant independent prognostic factor (*P* = 0.034); younger patients had a shorter survival time. Consistent with our findings, both Nemoto el at [9]. and Jingu el at [29].reported that younger patients had a worse prognosis. Biological behavior of tumors in younger patients may be aggressive. Thence, cancer screening is recommended to be early performed. In the present study, there were no other factors observed to be significantly associated with prognosis. Although some common prognostic factors including single lymph node recurrence, single regional lymph node recurrence, and small lymph node have been reported to be associated with better prognosis [10, 12, 14, 30].

The survival rate was not significantly different for a radiation dose that was higher or less than 60Gy (*p* = 0.888), although patients who received a higher dose had a longer survival time (median OS of 21 months) than those who received a lower dose (median OS of 16 months). In our case, all patients accepted intensity modulated radiotherapy, which could possibly be the reason for our encouraging finding. Raoul et al. [31] reported a median survival time of 10.7 months in patients combined chemotherapy with cisplatin and 5-FU with an RT dose of 60Gy. The low survival rate was associated with factors such as 2DCRT radiotherapy and multiple region recurrences. Kawamoto et al. [12] reported a favorable result with the median OS of 22 months after using a radiation dose of 60-66Gy. Given the advancements in radiotherapy technology, Zhang et al. [23] showed that doses higher than 60Gy significantly improved the progression-free survival and overall survival (median OS of 16.3 months, *p* = 0.041). However, due to the small size of their sample, and the lack of multivariate analysis, their findings may have been affected by bias. In the study run by Ma et al. [10], 98 patients received a radiation dose of 62-70Gy. They reported exciting finding of a median OS of 35 months in the concurrent chemotherapy group and that of 19 months in the radiotherapy group. Despite the numerous reported dose-related studies so far, the optimal dose for local recurrence after esophageal cancer surgery is yet to be agreed on. A high dose above 60Gy may be suitable with no associated patient deaths.

The present study has several limitations associated with its retrospective design. This study could not demonstrate a survival benefit between salvage RT and CCRT compared to the previous reports. In the present study, various chemotherapy regimens were used, but platinum or fluorouracil monotherapies were mainly used. Recent studies have suggested the TP or FP regimens can increase the survival time of recurrent esophageal cancer [12, 21, 29]. There were some selection biases: small sample size, the selection of primary therapy was different among surgeons, as well as the heterogeneity of the combined chemotherapy regimen, recurrent time, RT dose and RT technology.

## Conclusion

Our results suggest that radiotherapy with or without concurrent chemotherapy is an effective and feasible salvage treatment for postoperative lymph node recurrence after occurrence of esophageal squamous cell carcinoma. In view of the shortcomings aforementioned, further research is necessary to identify recurrent esophageal cancer in patients who should be treated with radiotherapy or chemoradiotherapy.

## Data Availability

The datasets used and/or analysed during the current study are available from the corresponding author on reasonable request.

## Abbreviations

3DCRT: Three-dimensional conformal radiation therapy
CCRT: Concurrent chemoradiotherapy
CR: Complete remission
CTV: Clinical target volume
FP: 5-fluorouracil plus platinum
GTV: Gross tumor volume
KPS: Karnofsky Performance Status
OS: Overall survival
PD: Progressive disease
PR: Partial remission
PTV: Planning tumor volume
RT: Radiotherapy alone
SD: Stable disease
TP: Paclitaxel plus platinum
VMAT: Volumetric modulated arc therapy
